# Groundwater discharge impacts marine isotope budgets of Li, Mg, Ca, Sr, and Ba

**DOI:** 10.1038/s41467-020-20248-3

**Published:** 2021-01-08

**Authors:** Kimberley K. Mayfield, Anton Eisenhauer, Danielle P. Santiago Ramos, John A. Higgins, Tristan J. Horner, Maureen Auro, Tomas Magna, Nils Moosdorf, Matthew A. Charette, Meagan Eagle Gonneea, Carolyn E. Brady, Nemanja Komar, Bernhard Peucker-Ehrenbrink, Adina Paytan

**Affiliations:** 1 University of California at Santa Cruz, 1156 High St., Santa Cruz, CA 95064 USA; 2 GEOMAR Helmholtz Center for Ocean Research, Wischhofstrasse 1-3, 24148 Kiel, Germany; 3 Princeton University, Princeton, NJ 08544 USA; 4 Woods Hole Oceanographic Institution, Woods Hole, MA 02543 USA; 5 Czech Geological Survey, Geologická 6, 152 00 Praha 5-Barrandov, Praha, Czech Republic; 6 Leibniz Centre for Tropical Marine Research (ZMT), Fahrenheitstraße 6, 28359 Bremen, Germany; 7 Kiel University (CAU), Christian-Albrechts-Platz 4, 24118 Kiel, Germany; 8 U. S. Geological Survey, 384 Woods Hole Rd, Woods Hole, MA 02543 USA; 9 University of Hawai’i at Manoa, 2500 Campus Rd, Honolulu, HI 96822 USA

**Keywords:** Element cycles, Geochemistry, Marine chemistry, Hydrology, Marine chemistry

## Abstract

Groundwater-derived solute fluxes to the ocean have long been assumed static and subordinate to riverine fluxes, if not neglected entirely, in marine isotope budgets. Here we present concentration and isotope data for Li, Mg, Ca, Sr, and Ba in coastal groundwaters to constrain the importance of groundwater discharge in mediating the magnitude and isotopic composition of terrestrially derived solute fluxes to the ocean. Data were extrapolated globally using three independent volumetric estimates of groundwater discharge to coastal waters, from which we estimate that groundwater-derived solute fluxes represent, at a minimum, 5% of riverine fluxes for Li, Mg, Ca, Sr, and Ba. The isotopic compositions of the groundwater-derived Mg, Ca, and Sr fluxes are distinct from global riverine averages, while Li and Ba fluxes are isotopically indistinguishable from rivers. These differences reflect a strong dependence on coastal lithology that should be considered a priority for parameterization in Earth-system models.

## Abstract (web summary)

Groundwater discharge is a mechanism that transports chemicals from inland systems to the ocean, but it has been considered of secondary influence compared to rivers. Here the authors assess the global significance of groundwater discharge, finding that it has a unique and important contribution to ocean chemistry and Earth-system models.

## Introduction

Globally, groundwater-derived solute fluxes, relative to riverine-derived fluxes, have been difficult to constrain due to large uncertainties in the volumetric flux, significant quantities of recirculated seawater, and chemical heterogeneity within—and between—aquifers. Volumetric flux estimates of global meteoric groundwater discharge vary by an order of magnitude, equivalent to 0.7–6% of global river discharge^1–5^. Despite this large uncertainty, several studies have attempted to constrain the magnitude and isotopic composition of groundwater-derived solute fluxes to the ocean using available data from local characterizations of groundwater chemistry^2,4,6–9^. These studies found that groundwater discharge may significantly impact the residence times of important nutrients in the ocean (e.g., N, P, and Si)^2,6,7^, serve as a net carbon sink in short- and long-term carbon cycles^9^, and can differ, isotopically, from riverine discharge (e.g., Sr and Nd)^4,8^. Quantifying and characterizing this terrestrially derived solute flux is, thus, central to our understanding of the sensitivity and interpretation of numerous oceanographic tracers, such as those relevant to silicate weathering and carbon cycling.

Recent models of global weatherability have emphasized the importance of silicate weathering in humid, low-latitude, tectonically-active regions^10,11^—the same regions modeled to account for the majority (55–68%) of global groundwater discharge^1,2,4^. This spatial overlap suggests that groundwater discharge may play an outsized role in terrestrially derived solute fluxes of silicate weathering products and the regulation of atmospheric CO_2_ on geologic timescales. However, groundwater-derived fluxes for major components (Ca and Mg) and trace element proxies (Li, Sr, and Ba) of weathering remain under-constrained^12–14^. This lack of constraint represents a potentially large source of uncertainty in the marine isotope budgets of δ^7^Li, δ^26^Mg, δ^44/42^Ca, ^87^Sr/^86^Sr, δ^88/86^Sr, and δ^138^Ba, as well as the Earth-system models reliant on their accuracy.

**Fig. 1:**
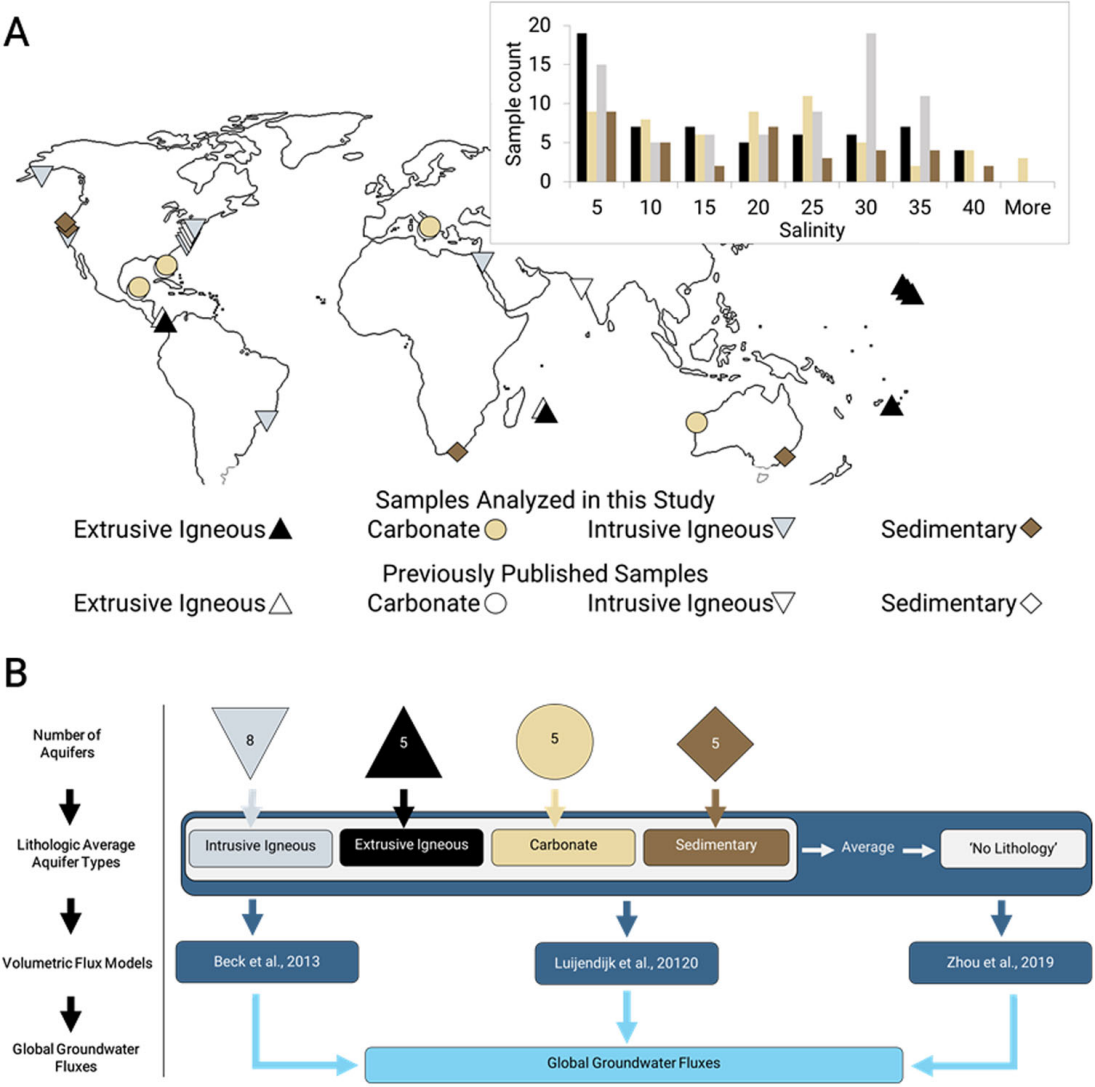
Overview of study design. **a** Map of aquifer locations (*n* = 27) from which data were utilized and an inset bar chart showing the number of samples from each salinity range used in this study (*n* = 229; this study and previously published data). **b** Flow diagram summarizing the calculation pathway to global groundwater-derived solute fluxes.

Here we test the hypothesis that, globally, groundwater discharge is a non-negligible (>1% of riverine discharge) source of Li, Mg, Ca, Sr, and Ba to the ocean and that the isotopic composition of its flux is distinct from riverine values for δ^7^Li, δ^26^Mg, δ^44/42^Ca, ^87^Sr/^86^Sr, δ^88/86^Sr, and δ^138^Ba. To test this hypothesis, we analyzed the concentration and isotope ratios of these solutes in groundwaters from 20 globally distributed subterranean estuaries^15^, in addition to incorporating previously published data from other coastlines^4,16,17^ (Fig. 1). This data was used to characterize geochemical endmembers of groundwater discharge from aquifers of four lithologic units. These endmember values were then fed into three independent, lithologically-weighted models of global groundwater discharge to estimate groundwater-derived solute fluxes to the ocean for each of these elements^1,2,4^. The resultant fluxes represent, at a minimum, 5% of global riverine flux values, which suggests that terrestrially derived solute flux estimates of these elements should be upwardly revised. Furthermore, the isotopic compositions of the global groundwater fluxes for these elements are not always identical to global riverine values.

## Results and discussion

### Coastal groundwater chemistry

**Fig. 2:**
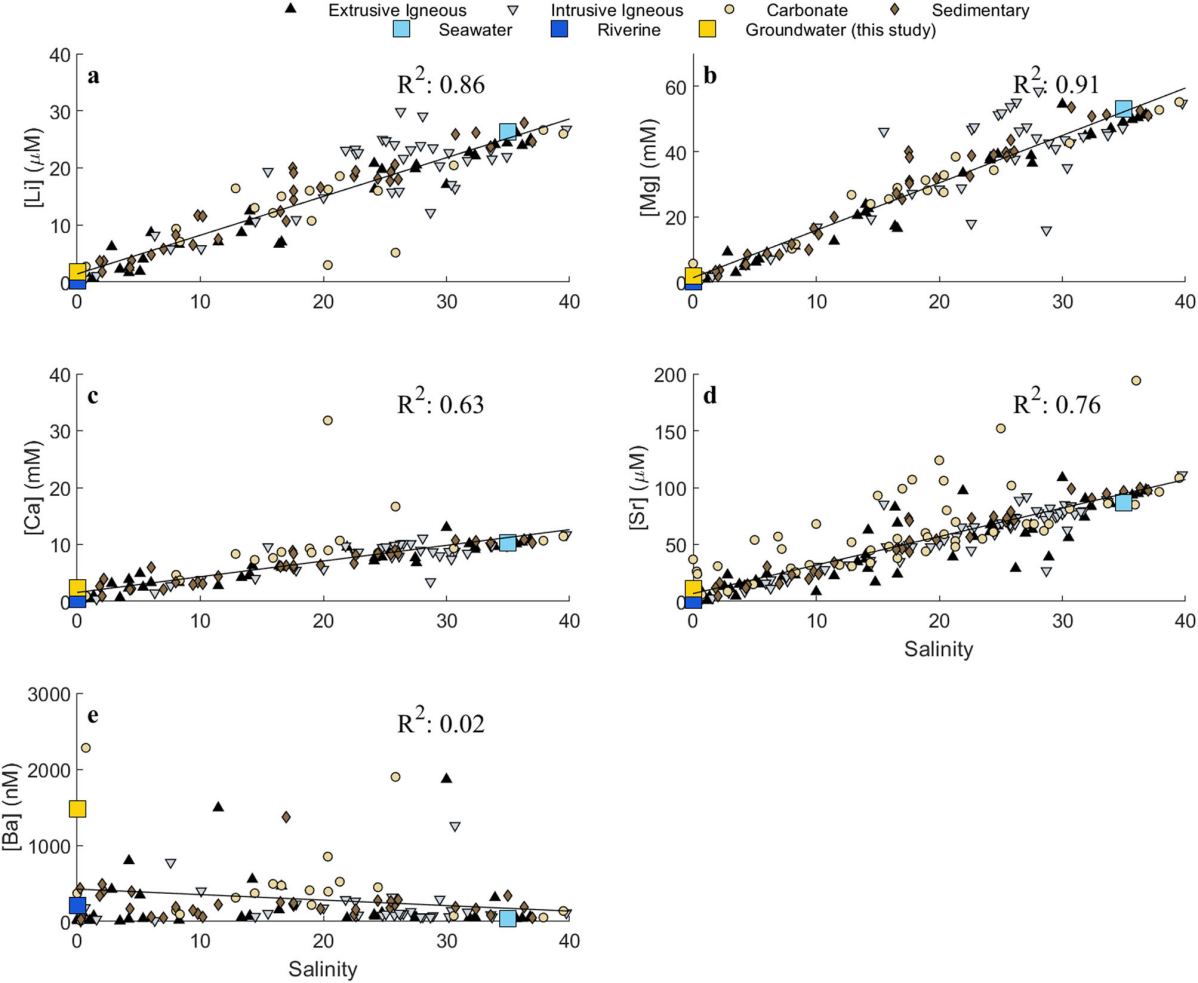
Elemental concentrations in samples. **a**–**e** Cation concentrations with respect to salinity, organized by lithologic aquifer type, plotted alongside relevant endmember values: seawater and global average riverine endmembers (references available in Table 1), as well as the average global groundwater composition calculated in this study. Linear regressions across all the data are plotted for each element to provide context of what conservative (Li, Mg, Ca, and Sr) vs. non-conservative (Ba) mixing across the salinity gradients would appear as. Sample sizes (*n*) are provided here: **a** [Li]: (*n* = 136), **b** [Mg]: (*n* = 139), **c** [Ca] (*n* = 140), **d** [Sr] (*n* = 225), **e** [Ba] (*n* = 56).

**Fig. 3:**
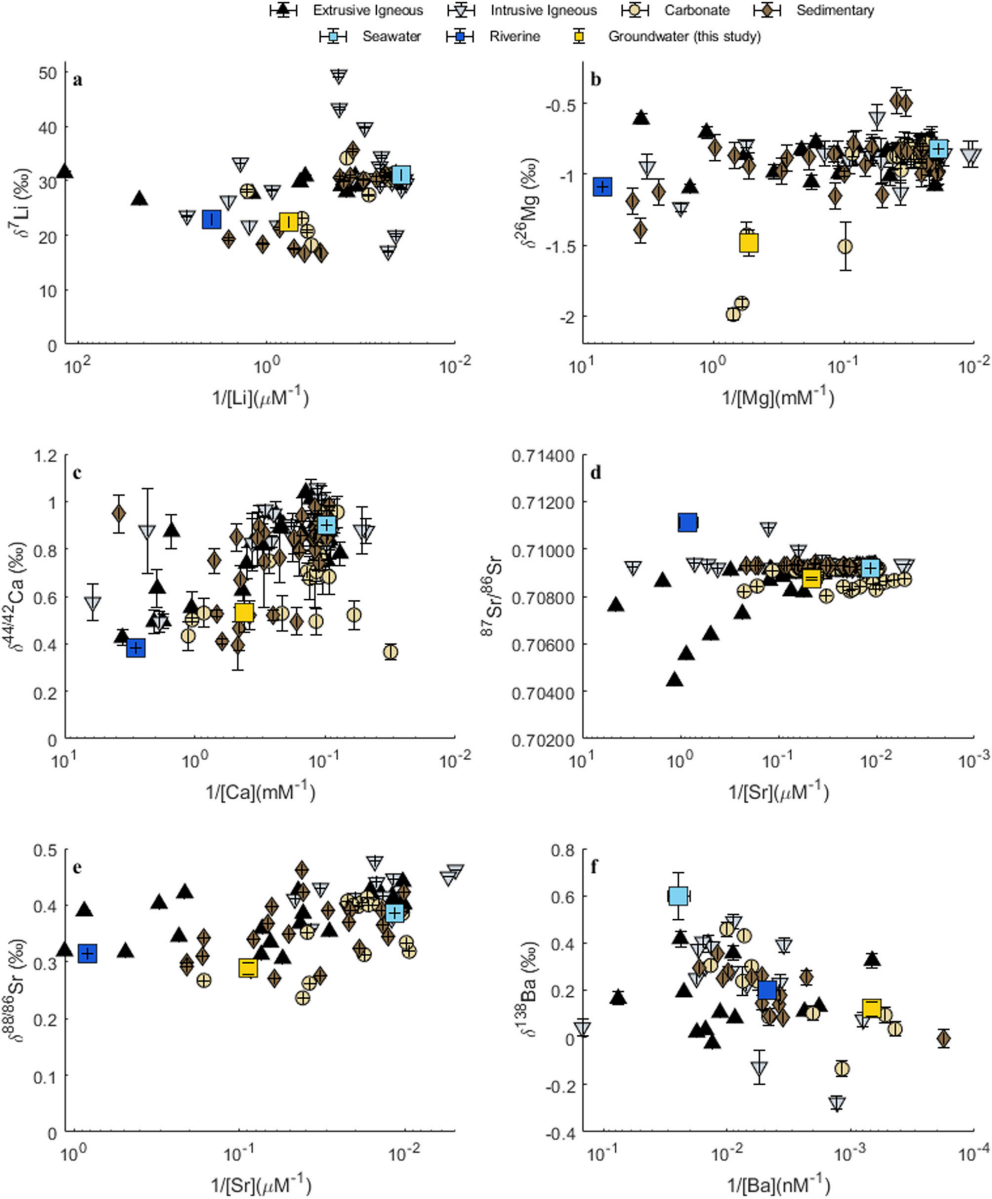
Isotopic composition of samples. **a**–**e** Isotopic compositions with respect to the inverse of cation concentrations, organized by lithologic aquifer type, plotted alongside relevant endmember values: seawater and global average riverine endmembers (references available in Table 1), as well as the average global groundwater composition calculated in this study. Error bars represent the greater of 2 standard deviations (2 SD) for each sample’s analysis or the long-term uncertainty (2 SD) of relevant standard reference materials discussed in the supplementary information. Sample size (*n*) values are provided here: **a** δ^7^Li (*n* = 67), **b** δ^26^Mg (*n* = 105), **c** δ^44/42^Ca (*n* = 114), **d** ^87^Sr/^86^Sr (*n* = 168), **e** δ^88/86^Sr (*n* = 79). **f** δ^138^Ba (*n* = 56).

The behavior of Li, Mg, Ca, and Sr concentrations in this collection of groundwater samples was conservative—representative of linear mixing between fresh, meteoric groundwater and saline, local seawater (Fig. 2, *R*^2^ values of linear trendlines across salinity gradients were >0.6). The concentrations of these elements in meteoric groundwater were approximately an order of magnitude more dilute that local seawater values, which caused the isotope ratios in the groundwater samples from higher salinities (>7) to be indistinguishable from seawater values (Fig. 3; Supplementary Table 4). However, the freshest groundwater samples did maintain unique isotopic signatures that, when extrapolated globally, plotted between average riverine and seawater values with respect to isotopic composition and the inverse of concentrations (Fig. 3).

In contrast, Ba exhibited non-conservative behavior in the subterranean estuaries due to its mid-salinity desorption maxima (Fig. 2)^18,19^. At these desorption maximum points, Ba concentrations in groundwater were up to an order of magnitude greater than local seawater (Fig. 2; Supplementary Table 3). These high concentrations, relative to seawater, resulted in predominantly distinct isotope ratios in the groundwater samples, relative to seawater, regardless of salinity (Fig. 3).

### Global groundwater fluxes

Since we define groundwater discharge in this study as the flow of meteoric waters directly from aquifers to the coastal ocean, only global groundwater discharge models specific to freshwater inputs were used^1,2,4^. The volumetric flux estimates of these groundwater discharge models used for extrapolation span 286–2400 km^3^ a^−1^ (Table 1)^1,2,4,5^, the resultant solute fluxes span a similarly large range. For example, in the case of Ba, the groundwater-derived solute flux ranges from 0.4 to 3.6 Gmoles a^−1^, which corresponds to 6–57% of the riverine flux (Table 1)^20^. Groundwater-derived flux estimates for Li, Mg, Ca, and Sr yield a similar, order of magnitude range (Table 1). All model outputs agree, however, that groundwater-derived solute fluxes of these elements represent, at a minimum, 5% of the riverine fluxes (Table 1).

**Table 1:** Chemical composition and fluxes.

Model	Beck et al., 2013	Zhou et al., 2019	Luijendijk et al., 2020	Riverine	Ref.
Volumetric Flux (km^3^ yr^−1^)	2,400	489	286	39,000	^3^
[Li] (μM)	1.8	1.7	1.7	0.26	^12^
Li (gigamoles yr^−1^)	4.3	0.9	0.5	10	
% of riverine flux	43%	9%	5%	—	
δ^7^Li (‰)	22.7	22.3	22.0	23	
[Mg] (mM)	1.7	1.9	2.0	0.14	^22^
Mg (teramoles yr^−1^)	4.2	0.9	0.6	5.6	
% of riverine flux	75%	16%	10%	—	
δ^26^Mg (‰)	−1.47	−1.48	−1.50	−1.09	
[Ca] (mM)	2.22	2.40	2.60	0.36	^23^
Ca (teramoles yr^−1^)	5.3	1.2	0.7	13.7	
% of riverine flux	39%	9%	5%	—	
δ^44/42^Ca (‰)	0.52	0.53	0.54	0.39	
[Sr] (μM)	10.1	11.4	12.6	3.21	^24^
Sr (gigamoles yr^−1^)	24.3	5.6	3.6	47	
% of riverine flux	52%	12%	8%	—	
^87^Sr/^86^Sr	0.70876	0.70874	0.70873	0.71106	^24^
δ^88/86^Sr (‰)	0.290	0.289	0.287	0.315	^30^
[Ba] (μM)	1.5	1.5	1.5	0.2	^20,21,26,27^
Ba (gigamoles yr^−1^)	3.6	0.7	0.4	6.3	
% of riverine flux	57%	11%	6%	—	
δ^138^Ba (‰)	0.12	0.12	0.12	0.16	

Model outputs for the chemical composition of global average groundwater discharge, given the same geologic aquifer endmember values. Riverine values from the denoted literature (final column) are reported for context. Solute concentrations from these references were converted to fluxes by multiplying reported concentrations by the global volumetric flux of riverine discharge, cited here (top row). No δ^138^Ba value for the global riverine flux has yet been published, so one is calculated in this study with all available data to date. Further discussion on this can be found in the SI. δ^44/42^Ca values can be converted to conventional δ^44/40^Ca values by multiplying by 2.05^35^.

Of the three global groundwater flux models used in this study, the two published most recently by Zhou et al.^1^ and Luijendijk et al.^2^ agreed more closely with one another and are in contrast with the earlier model by Beck et al.^4^. However, there is still almost a two-fold difference in groundwater discharge volumes (286 vs. 489 km^3^ a^−1^) between the two recent models^1,2^. This range in global estimates of groundwater discharge limits our ability to precisely estimate solute fluxes, but the geochemical data reported here can be used to update estimates of groundwater-derived solute fluxes once new estimates of discharge become available. Additionally, new chemical data collected from subterranean estuaries in under-sampled regions, particularly in tropical regions with high groundwater discharge, would help to better constrain the endmember concentrations and isotope ratios used in this study.

### Isotopic compositions of global groundwater fluxes

Compared to the large uncertainties of groundwater-derived solute flux estimates, the isotopic compositions of these fluxes are in closer agreement with each other (Table 1). The isotopic compositions of the global groundwater flux were calculated as the average of the two model outputs for which lithologic weighting was available^2,4^. This resulted in the isotopic compositions of modern groundwater discharge to be determined as 22.3 ± 0.7‰ (δ^7^Li), −1.40 ± 0.09‰ (δ^26^Mg), 0.53 ± 0.08‰ (δ^44/42^Ca), 0.70878 ± 0.00005 (^87^Sr^/86^Sr), 0.292 ± 0.010‰ (δ^88/86^Sr), and 0.12 ± 0.03‰ (δ^138^Ba), where the uncertainties represent the greater of model output ranges or long-term analytical uncertainty (Table 1; see Supplementary Methods for additional details on standard reference materials). The δ^7^Li and δ^138^Ba compositions of the global groundwater flux to the ocean, based on our data and model results, are nearly identical to average riverine values^12,21^ (Table 1). However, the δ^26^Mg, δ^44/42^Ca, δ^88/86^Sr, and ^87^Sr^/86^Sr values are distinct from average riverine compositions^22–24^ (Table 1).

### Interpreting the heterogeneity of global groundwater fluxes

The global groundwater fluxes and isotopic compositions of the suite of elements reported here heavily depend on the distribution of coastal lithology in the models and the geochemical data obtained from each of these distinct lithologies. All estimates of global groundwater discharge agree that the majority (55–68%) of discharge is derived from tectonically active, tropical regions^1,2,4^, which are typically characterized by young volcanic rocks and coastal carbonate deposits, hence skewing the global average towards these lithologies. The global riverine flux, in contrast, is more evenly distributed latitudinally and the chemistry of river discharge is more representative of the average exposure of different rock types on land^24^.

We argue that this is the cause of the observed differences (Mg, Ca, Sr) or similarities (Li, Ba) calculated between the isotopic compositions of riverine and groundwater-derived solute fluxes. The dominance of young volcanic rocks and recent carbonate deposits (e.g. paleo-reefs) in low latitude areas with high groundwater discharge contribute to the less radiogenic ^87^Sr^/86^Sr composition of the global groundwater flux, relative to riverine discharge. This finding is consistent with a previous assessment of global groundwater ^87^Sr^/86^Sr composition^4^ and in situ investigations of groundwater-derived Sr fluxes from these regions^10,25^. In addition to a less radiogenic ^87^Sr^/86^Sr composition, coastal carbonate deposits also have an abundance of Mg, Ca, and Sr with lower δ^26^Mg, δ^44/42^Ca, and δ^88/86^Sr values, relative to seawater, as reflected in our data. The abundance of coastal carbonate deposits in tropical regions disproportionally controls the δ^26^Mg, δ^44/42^Ca, and δ^88/86^Sr signatures of global groundwater discharge, resulting in lighter signatures distinct from riverine fluxes of these elements (Table 1).

We attribute the isotopic similarity between groundwater and riverine-derived fluxes of Li and Ba to inherent characteristics that make them less susceptible to differences in geologic age of substrate and coastal carbonate deposits. The δ^7^Li composition of groundwater derived from extrusive igneous lithologies was indistinguishable from that of intrusive igneous lithologies (Table 2). Furthermore, since Li is not readily incorporated into carbonates and the δ^7^Li composition of groundwater derived from carbonate aquifers was similar to that of igneous aquifers (Table 2), the abundance of coastal carbonate deposits in regions of high groundwater discharge had minimal impact on the Li isotopic composition of the global groundwater flux. Similarly, the Ba isotopic composition of groundwater derived from each of the lithologic aquifer endmembers exhibited a narrow range (0.14‰) (Table 2). For example, the carbonate aquifer endmember had a δ^138^Ba value of 0.09‰ and the extrusive igneous value was 0.18‰, which are both similar to the global riverine average (δ^138^Ba ≈ 0.2 ± 0.1‰)^21,26,27^. The isotopic compositions of Ba in global river and groundwater fluxes (δ^138^Ba = 0.12 ± 0.03‰) are indistinguishable, but are both conspicuously heavier than Bulk Silicate Earth (≈0.0 ± 0.1‰)^28^. This offset is suggestive of a small, but systematic, isotopic fractionation during weathering or transport, such as the loss of isotopically light Ba precipitating into secondary minerals or adsorbing to sediments, respectively.

**Table 2:** Geologic aquifer type endmember compositions and model outputs of global groundwater chemistry.

Parameter Aquifer Type	[Li] _(μM)_	δ^7^Li _(‰)_	[Mg] _(mM)_	δ^26^Mg _(‰)_	[Ca] _(mM)_	δ^44/42^Ca _(‰)_	[Sr] _(μM)_	^87^Sr/^86^Sr	δ^88/86^Sr _(‰)_	[Ba] _(μM)_	δ^138/134^Ba _(‰)_
Extrusive Igneous	0.04	28.6	0.1	−0.85	1.1	0.51	3	0.70695	0.357	0.8	0.10
Carbonate	2.42	24.0	3.3	−1.78	4.3	0.49	15	0.70862	0.255	1.5	0.11
Intrusive Igneous	1.81	28.4	2.1	−1.09	0.8	0.53	2	0.71253	0.411	1.0	0.01
Sedimentary	1.52	19.1	0.7	−1.08	1.5	0.62	2	0.70931	0.354	1.6	0.15
No Lithology	1.45	25.0	1.6	−1.20	2.0	0.54	5	0.70935	0.344	1.3	0.10

No Lithology represents the Complex/No Litho classification units used in the Beck et al.^4^ and Luijendijk et al.^2^, respectively. It is represented by a concentration-weighted average of all four lithologic average aquifer type endmembers.

### Relevance to Earth-System models

**Fig. 4:**
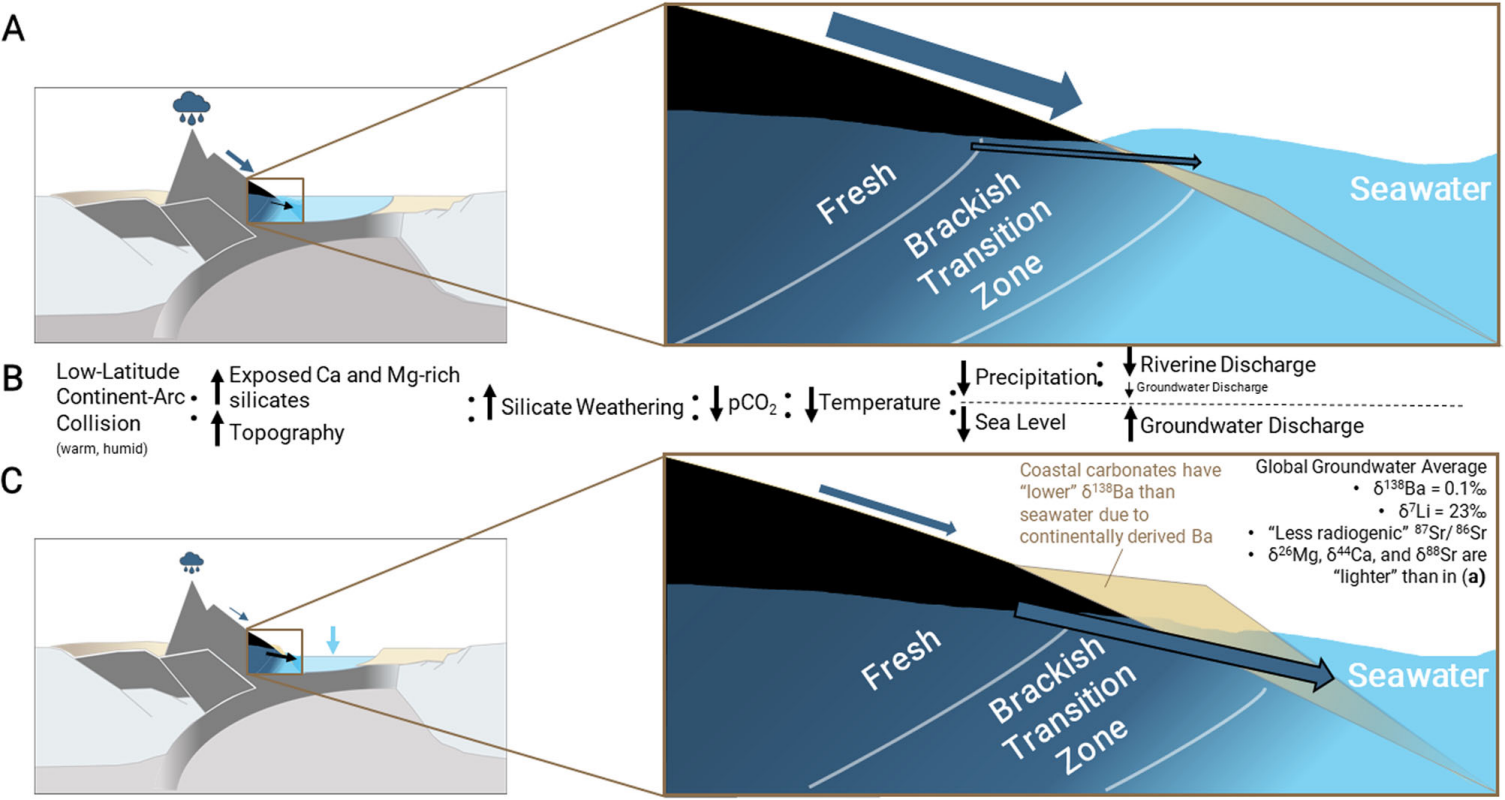
Illustration of a subterranean estuary during a shift to glacial conditions. **a** Interglacial stage steady-state conditions with the subterranean estuary magnified on the right. **b** Conceptual outline of the feedback outlined by Macdonald et al.^11^, where a low-latitude island arc collision instigates the onset of global cooling. **c** Glacial conditions with less precipitation and falling sea level with the subterranean estuary magnified on the right and a description of the chemical changes that might occur to the isotopic composition of global groundwater-derived solute fluxes. The tan triangle represents coastal carbonate deposits, whose δ^138^Ba composition may be lighter than seawater values if previously exposed to terrestrial (groundwater and river water) runoff.

The differences and/or similarities between riverine and groundwater discharge in solute composition should not be considered as static. We suggest that their solute fluxes may change over geologic timescales due to differing hydrologic controls on volumetric fluxes – namely, precipitation and changes in sea level^29,30^, as well as changes in the distribution of land masses and associated lithologies relative to climate regions. For example, during glacial periods, diminished precipitation would decrease riverine discharge^31^, while falling sea levels would transitionally increase groundwater discharge and exposure of carbonate shelves^13,29,30^. These conditions should, consequently, increase the relative importance of groundwater-derived solute contributions globally and its associated isotopic signatures (Fig. 4).

Reconstructing temporal and spatial changes in riverine and groundwater discharge, however, is a difficult task. Geochemical proxies, such as Ba/Ca ratios of coastal carbonate deposits have been utilized with some success^32^, based on the premise that Ba concentrations of terrestrial water fluxes are high (≥100 nM) relative to surface seawater (generally < 50 nM), thus elevating the Ba/Ca ratio of near-shore seawater, and resulting in a higher Ba/Ca ratio in coastal carbonate deposits. In coastal areas influenced by riverine or groundwater discharge, the Ba incorporated into these carbonate deposits should also possess lower δ^138^Ba than that of seawater (+0.3 to +0.6‰)^14^, more similar to the composition of meteoric waters found in this study and the global riverine average (≈ +0.2‰)^21,27^. This implies that Ba isotopes may have utility as a complementary paleo-proxy to Ba/Ca ratios for the reconstruction of terrestrial water input fluctuations in nearshore deposits to estimate changes in the relative influence of terrestrial water inputs over time.

### Conclusions

This study shows that groundwater-derived solute fluxes of Li, Mg, Ca, Sr, and Ba are of significance to marine isotope budgets, since solute fluxes amount to at least 5% of the riverine input. Moreover, the isotopic compositions of some of these solutes are distinct from those of riverine discharge. This additional solute flux impacts mass balance models of these, and possibly other, elements, as well as their isotopic compositions in seawater. Due to the reliance on modeled volumetric fluxes of global groundwater discharge, as well as the limited number and wide range of volumetric estimates currently available, we deliberately report our results as minimum flux estimates. As additional models and geochemical data become available, these fluxes should be refined, though it is already clear that groundwater-derived solute fluxes are non-negligible and warrant inclusion in marine isotope budgets.

## Methods

### Dataset sources

Samples were opportunistically amassed from groundwater researchers worldwide (Supplementary Table 2). In the field, these samples were collected using traditional groundwater-sampling techniques—namely, digging pits or inserting piezometers into beach faces perpendicular to the shoreline to obtain a salinity-gradient: from fresh, meteoric groundwater to local coastal seawater (Supplementary Fig. 1). Post-collection, samples were filtered through either 0.2 or 0.45 μm filters and acidified. Samples were in storage for anywhere between a few weeks to 9 years before analysis. Given the consistency of cation concentrations in the samples independent of storage time, and the order-of-magnitude variability in the model outputs when extrapolating the data globally, evaporation during storage is negligible.

Due to the opportunistic nature of this sampling, sub-categories within each lithologic aquifer type are not evenly represented. For example, the extrusive igneous category is comprised predominantly of samples representing young lithologies (e.g., Hawai’i and Mauritius), while groundwater samples from older extrusive igneous formations (e.g., Deccan Trap region) are not represented. This may have influenced the representativeness of the dataset; for example, the ^87^Sr/^86^Sr composition calculated for the extrusive igneous member might be more radiogenic than we have calculated here. Most importantly, affluent regions are overrepresented and developing regions, such as Indonesia and western Africa, which account for a large portion of the global groundwater flux, were not sampled. We argue, however, that it is coastal carbonate deposits that are the main drivers of isotopic differences between groundwater and riverine discharge globally, and that these are well-characterized by this study, since most coastal carbonate deposits should be relatively recent in age. Of course, improvements to the globally representative nature of this sample set are desired.

### Historical data incorporation

Published data from other coastal aquifers/subterranean estuaries that included at least one of the isotope systems relevant to this study were included in the meteoric groundwater endmember calculations (Supplementary Table 2)^4,16,17^. Data from non-coastal aquifers were not included because the focus of this study was specifically to examine the groundwater-derived solute flux to the ocean and it is unclear if and how much transformation these constituents experience in the aquifer before reaching the coast. Thus, to ensure that data represent the solute load transported to the coast, only coastal groundwater samples and data from coastal subterranean estuaries were included.

### Chemical analyses

Dissolved concentrations of Li, Mg, Ca, Sr, and Ba were analyzed at the University of California, Santa Cruz using a Thermo Scientific^TM^ Element XR^TM^ inductively coupled plasma mass spectrometer (ICP-MS). Subsampling and subsequent analyses of dissolved concentrations to ensure reproducibility were performed at the Woods Hole Oceanographic Institution and the Czech Geological Survey with a Thermo Scientific iCAP Qc quadrupole ICP-MS and Agilent 5110 ICP-optical emission spectrometer, respectively. The δ^7^Li, δ^26^Mg, δ^44/42^Ca, and δ^138/134^Ba data were collected using a Thermo Scientific^TM^ Neptune^TM^ multi-collector ICP-MS at the Czech Geological Survey, Princeton University, GEOMAR Helmholtz Center for Ocean Research, and Woods Hole Oceanographic Institution, respectively. Strontium isotope data (^87^Sr^/86^Sr and δ^88/86^Sr) were obtained via thermal ionization mass spectrometry at GEOMAR. All isotope data, except ^87^Sr/^86^Sr, are reported in per-mil (‰) relative to the corresponding reference materials using delta (δ) notation. Additional information on technical aspects of the analyses and a complete tabular compilation of the data is available in the Supplementary Information.

### Lithologic aquifer type characterizations

Five lithologic aquifer types were designed for use in the lithologically weighted groundwater discharge models. These types were: extrusive igneous, intrusive igneous, carbonate, sedimentary, and ‘no lithology.’ Individual aquifers (*n* = 27) were categorized into one of these four geologic aquifer types and these categorizations are listed in Table 2. These categorizations were assigned based on descriptions of local geology from the papers that conducted the initial sampling/study or, for uncommon instances where geology was not defined in the sampling, regional maps of geology were used.

In the two global groundwater discharge models with lithologic weighting, by Beck et al.^4^ and Luijendjik et al.^2^, there are instances where the local geology of aquifers is entirely or partially unconstrained, which they respectively refer to as ‘complex’ and ‘no lithology.’ Since this unconstrained aquifer type accounts for 37%^4^ and 10%^2^ of the global volumetric fluxes presented in the respective models, it was necessary to estimate a chemical composition for this endmember. Therefore, we designed a fifth ‘no lithology’ aquifer type, whose elemental concentration was equal to the average of the other four lithologic aquifer types and whose isotopic composition was calculated via a concentration-weighted average of the other four aquifer types (Fig. 1; Table 2).

### Geochemistry of lithologic endmembers

Elemental concentrations across the salinity gradient of the subterranean estuaries analyzed in this study were evaluated for conservative vs non-conservative behavior. The behaviors of Li, Mg, Ca, and Sr concentrations were conservative (Fig. 2, *R*^2^ values of linear trendlines across salinity gradients were >0.6) and, thus, their freshwater endmember concentrations were estimated to equal the zero-salinity intercept of linear regressions for each aquifer type (Table 2). By using the intercept, which represents cation concentrations at zero salinity, we avoid overestimating the solute concentrations in fresh groundwater discharge due to recirculating seawater, whose influence is not within the scope of this study. Barium exhibited non-conservative behavior in our dataset, with maximum Ba concentrations ([Ba]_max_) occurring at intermediate salinities, consistent with the expected mid-salinity desorption maxima of Ba in subterranean and riverine estuaries (Fig. 2)^18,19^. The [Ba]_max_ value for each aquifer was used to represent the groundwater endmember, since desorbed Ba from coastal sediments is presumed to be a new (i.e., non-recirculated) source of Ba to the ocean, indicating that the [Ba]_max_ value more accurately represents the concentration in the groundwater as it discharges to the ocean. Using the freshwater endmember, derived from the intercept, would have underestimated the groundwater-derived Ba flux. Thus, the Ba concentration in the groundwater endmember was estimated to equal the average of [Ba]_max_ samples within each aquifer type (Table 2).

The δ^7^Li, δ^26^Mg, δ^44/42^Ca, ^87^Sr/^86^Sr, and δ^88/86^Sr compositions of each aquifer type endmember were calculated as the average of the corresponding isotope compositions from the lowest salinity samples in each aquifer, provided that the sample had a salinity <2 (Fig. 3; Table 2). Using only isotopic compositions of low-salinity samples reduced the likelihood of unintentionally incorporating values influenced by recirculated seawater into the groundwater flux characterization. The δ^138^Ba composition of each aquifer type was calculated as the average of the values from each of the [Ba]_max_ samples (Fig. 3; Table 2). Since little is known about the role that desorption from coastal sediments plays in Ba isotope fractionation, it was prudent to only use the isotope values of the samples from which the cation flux was derived.

### Global extrapolation

The results of this geochemical survey of coastal groundwaters were extrapolated globally using three volumetric groundwater fluxes: Beck et al.^4^, Luijendijk et al.^2^, and Zhou et al.^1^. The first two of these models were lithologically weighted^2,4^; meaning that volumetric flux values for each groundwater region had a distribution of lithology associated with it (e.g., 40% carbonate, 60% sedimentary, etc.). Thus, the resultant flux from that region would have a chemical composition equal to the proportionate mixture of elemental concentrations and isotope ratios from those aquifer type endmembers characterized in this study.

#### Beck et al., 2013 Model

The model developed by Beck et al.^4^ is based upon the volumetric groundwater fluxes of Zektser^5^ and the geologic map of Gibbs & Kump^33^. This Zektser model delineates 118 ‘groundwater regions,’ for which volumetric fluxes of groundwater are calculated. This volumetric model was adapted by Beck et al.^4^ to include lithologic weighting by overlaying a coarse (2° x 2°) geologic map by Gibbs & Kump^33^. In the Beck et al.^4^ model, there was an additional aquifer type for slate lithologies that was removed in this study due to a lack of samples derived from slate-dominated aquifers. Before the addition of this new data, however, the slate aquifer endmember was removed and there was no change to the resultant Sr flux or isotopic composition calculated with the data from Beck et al. (2013). We adapted the model, however, to include solute flux-weighting to the isotopic composition of the global output.

In the original model, an unweighted average Sr concentration ([Sr]) and ^87^Sr/^86^Sr value was used for the composition of all 118 ‘groundwater regions.’ However, in this study, the elemental flux (i.e., concentration * annual groundwater flux) for each of the 118 ‘groundwater regions’ was used to calculate the isotopic composition of the global groundwater-derived solute fluxes. The calculations were as follows:

1. Calculate annual solute fluxes (*f*_*n*_) for each of the 118 groundwater regions:
  $$f_n = \left( {\left[ X \right]_n^\ast Q_n} \right)$$
  where *[X]*_*n*_ refers to the solute concentration in a ‘groundwater region,’ which is dictated by local geology, and *Q*_*n*_ refers to the volumetric discharge of groundwater from that region.
2. Calculate % of global groundwater-derived flux for each ‘groundwater region’ (p):
  $${\mathrm{p}}_{\mathrm{n}} = \frac{{{\mathrm{f}}_{\mathrm{n}}}}{{\mathop {\sum }\nolimits_{{\mathrm{i}} = 1}^{118} {\mathrm{f}}_{\mathrm{i}}}}$$
  where the flux (*f*) from an individual groundwater region (*n*) is divided by the summation of fluxes from all 118 global ‘groundwater regions.’
3. Calculate solute concentrations for global groundwater discharge ($Groundwater_{avg}\left[ X \right]$):
  $$Groundwater_{avg}\left[ X \right] = \mathop {\sum }\limits_{i = 1}^{118} p_i^\ast \left[ X \right]_i$$
  where $\left[ X \right]_i$ represents the solute concentration for each aquifer.
4. Calculate isotopic compositions for global groundwater discharge ($Groundwater_{avg}\delta X$):
  $$Groundwater_{avg}\delta X = \frac{{\mathop {\sum }\nolimits_{m = 1}^{118} \delta X_m^\ast f_m}}{{\mathop {\sum }\nolimits_{i = 1}^{118} f_i}}$$
  where ${\updelta}X_m$ represents the isotopic composition of a solute for global groundwater discharge, *f*_*i*_ denotes the solute flux from each ‘groundwater region,’ and *f*_*m*_ is the global solute flux, namely: $Groundwater_{avg}\left[ X \right]$ multiplied by the volumetric estimate of global groundwater discharge from each model.

This change to the calculation of the chemical composition of global groundwater discharge only resulted in a small difference relative to the original model^4^ in the final output. For example, the Li concentration in the global groundwater flux changed from 1.6 to 1.8 µM and δ^7^Li changed from 24.0‰ to 23.1‰. These latter values (1.8 µM and 23.1‰), however, are more representative of the chemical composition of the global groundwater flux than the unweighted average because this solute flux-weighting approach corrects for the inequitable influence that regions responsible for only a small fraction of global groundwater discharge were having on the calculation of global groundwater chemistry. For example, in the original model, the chemical composition of Jamaica (a ‘groundwater region’ responsible for only 0.02% of the global volumetric flux and 0.04% of the global Ca flux) had the same impact on the global groundwater Ca isotopic composition as more voluminous ‘groundwater regions’ that are responsible for a greater portion of the global groundwater-derived Ca flux (e.g. New Guinea, which is responsible for 11% of the global volumetric flux and 4% of the groundwater-derived Ca flux). With this update to the model, each of these 118 ‘groundwater regions’ are not weighted equally to one another in the calculation of global groundwater chemistry, but rather were considered in proportion to their volumetric and solute fluxes.

#### Luijendijk et al., 2020 Model

This volumetric model of global groundwater discharge integrates the watershed geometry, topographic gradients, permeability, and groundwater recharge of 39,858 coastal watersheds to construct one, global-scale groundwater flux. Lithologic weighting is integrated in this model as 17 different lithologic groupings derived from the GLiM geologic map^34^. Without the ability to characterize the chemical composition of 17 distinct lithologic aquifer type endmembers in this study, these 17 lithologies were binned into the same five aquifer types shared by Beck et al.^4^—namely, extrusive igneous, intrusive igneous, carbonate, sedimentary, and ‘no lithology’ (Table 3). The relative abundance of each lithology within each ‘groundwater region,’ however, was far more precise in this model. Beck et al.^4^ estimated watershed geologic make-up as a binary combination (i.e., present vs. absent) of each lithology (e.g., an aquifer with carbonates, granites, and sedimentary rocks would always be represented by a 33.3% share of each—additional precision was not resolved). This model utilized a higher-resolution geologic map^34^, which yielded a higher-resolution estimation of watershed geology.

**Table 3:** Endmember classification.

GLiM Model Geologic Units	Aquifer Type Classification (this study)
va	Extrusive Igneous
vb	
vi	
pa	Intrusive Igneous
pb	
pi	
ig	
mt	
ev	Carbonate
sc	
sm	Sedimentary
ss	
su	
wb	
py	
Nolitho	‘No Lithology’
nd	

Geologic units from the GLiM map^34^, used in the Luijendijk et al.^2^ model, grouped into the five aquifer type endmembers used in this study.

#### Zhou et al., 2019 Model

This volumetric model of global groundwater fluxes is based on continental-scale hydrographic and climate datasets from NASA, spanning 60°N to 60°S. However, no lithologic weighting was considered in this model. In lieu of lithologic weighting, we opted to take the average of the final chemical compositions of global groundwater discharge calculated via the geologic weighting in the other two models: Beck et al.^4^ and Luijendijk et al.^2^ and simply multiply by the Zhou et al.^1^ flux.

## Supplementary information

Supplementary Information

## Data Availability

All datasets generated in this study can be found in the Supplementary Information to this study.
