# Supplementary material for: Groundwater discharge impacts marine isotope budgets of Li, Mg, Ca, Sr, and Ba
Source: Nat Commun. 2021 Jan 8;12:148. doi: 10.1038/s41467-020-20248-3 (PMC7794541; doi:10.1038/s41467-020-20248-3)
Supplement: Supplementary file 1 — Supplementary Information [file 41467_2020_20248_MOESM1_ESM.pdf]

Supplementary Information for

‘Groundwater Discharge Impacts Marine Isotope Budgets of Li, Mg, Ca, Sr, and Ba’

by Kimberley K. Mayfield, et al., 2020

Supplementary Notes

Riverine Ba Flux Characterization

Due to the novelty of the δ^{138}Ba system, no global riverine composition had yet been constrained. To construct a first-order approximation of this value, we compiled data from the three studies for which δ^{138}Ba was published and estimate a discharge-weighted [Ba] and δ^{138}Ba composition of global average river discharge (Supplementary Table 1).

Supplementary Methods

Cation Concentration Analyses

All samples (*n* = 134) were analyzed for solute concentrations at the Marine Analytical Laboratory at UCSC and a subset of these samples (*n* = 56) were analyzed at Woods Hole Oceanographic Institution (WHOI) using a ThermoFinnigan ELEMENT II sector-field ICP-MS and at the Czech Geological Survey (CGS) with an Agilent 5110 ICP-optical emission spectrometer (OES). The results of this inter-lab comparison (*n* = 51) yielded an agreement for all elements within 5%.

a. Li Isotope Analyses

Lithium analytical procedures and isotopic measurements were performed at the CGS using a method developed by Magna et al. (2004) [20], which involves ion-exchange chromatography with BioRad™ AG-50W-X8 cation exchange resin (repeated twice), followed by Li isotope analysis using a Neptune multi-collector inductively-coupled-plasma mass spectrometer (MC-ICP-MS; Thermo Scientific™). A sample-standard bracketing method using L-SVEC solution was utilized to determine natural Li isotopic variations in the groundwater

samples. The results of Li isotopic measurements are reported in the δ-notation relative to the L-SVEC reference solution [21] and calculated as:

$$\delta^7Li\ (‰) = [(^7Li/^6Li)_{sample} / (^7Li/^6Li)_{L-SVEC} - 1] \times 1000$$

Four different reference materials were repeatedly analyzed alongside samples to monitor the consistency of the entire procedure: IAPSO (OSIL seawater; 30.85‰ ± 0.18; n = 4), NASS-6 (seawater; NIST) (30.78‰ ± 0.08; n = 3), NIST 1640a (river water; NIST) (16.76‰ ± 0.08; n = 4), and SLRS-5 (river water; NRC) (23.58‰ ± 0.33; n = 3), where the uncertainty represents 2SD. The resultant δ^7Li values for the standards fell within the range of previously published values available on GeoRem [22] and their reproducibility between runs (± ≤ 0.33‰; 2SD) was approximately equal to the uncertainty associated with their individual analyses (0.07 – 0.54‰). All analytical results for the δ^7Li composition of groundwater samples are in the Supplementary Data Tables section.

b. Mg Isotope Analyses

Magnesium isotopic compositions were measured at Princeton University using a method developed by Blättler et al. (2015) [23], which involves automated, high pressure ion-exchange chromatography with a Dionex ICS-5000+ IC system, coupled with a Dionex AS-AP fraction collector, followed by Mg isotope analysis with a Neptune MC-ICP-MS (Thermo Scientific™). A sample-standard bracketing method using DSM-3 solution [24] was utilized to determine natural Mg isotopic variations in the groundwater samples. The results of Mg isotopic measurements are reported in δ-notation relative to the DSM-3 reference solution and calculated as:

$$\delta^xMg\ (‰) = [(^xMg/^{24}Mg)_{sample} / (^xMg/^{24}Mg)_{DSM-3} - 1] \times 1000$$

where the ‘x’ denotes 26 or 25, respectively.

To ensure that the separation procedure did not fractionate Mg, we processed standards alongside unknowns. Measured δ^{25}Mg and δ^{26}Mg compositions for IC-purified Cambridge-1 (δ^{25}Mg = -1.35 ±0.10‰; δ^{26}Mg = -2.62 ±0.13‰; 2σ, n=13) and Bermuda seawater (δ^{25}Mg = -0.45 ±0.09‰; δ^{26}Mg = -0.84 ±0.12‰; 2σ, n=13) standards are indistinguishable from published values [24], attesting to the efficacy of our chemical purification protocol.

Measured δ^{26}Mg and δ^{25}Mg were plotted (Supplementary Figure 2) and the trendline slope (0.532) was compared to previous studies [25] to ensure mass-dependent behavior of Mg isotope fractionation in this sample set. The slope in Supplementary Figure 2 is within analytical uncertainty of that published in Higgins & Schrag, 2010 [25], which was 0.527 ± 0.009. All analytical results for the δ^{26}Mg composition of groundwater samples are provided in Supplementary Data Tables. For replicated samples (chemical purification + mass spectrometry), we report the 2σ error on the sample average; otherwise, reported errors correspond to long-term external precision on the Cambridge-1 standard at Princeton (0.09‰, 2σ, n=79; Blattler et al., 2015).

c. Ca Isotope Analyses

Calcium isotopic compositions were measured at GEOMAR Helmholtz Center for Ocean Research (Kiel, GER). Chemical separation of Ca from the matrix was conducted using an automated, commercially-available Prep*FAST* MC (ESI, Omaha, NE, USA) according to the method developed by Romaniello et al., (2015) [26]. The $\delta^{44/42}$Ca analyses were conducted on a Neptune™ MC-ICP-MS (Thermo Scientific™) with a method adapted from Eisenhauer et al. (2019) [27], which utilizes a sample-standard bracketing technique using SRM-915a solution [28] to determine natural Ca isotopic variations. The results of Ca isotopic measurements are reported in δ-notation relative to the SRM-915a reference solution and calculated as:

$$\delta^{44/42}Ca\ (‰) = [(^{44/42}Ca/^{44/42}Ca)_{sample} / (^{44/42}Ca/^{44/42}Ca)_{SRM\text{-}915a} - 1] \times 1000$$

Three different reference materials were separated and analyzed alongside samples to monitor the consistency of the entire procedure: IAPSO (seawater; OSIL), SRM-915b (calcium carbonate; NIST), and SRM-1486 (bone meal; NIST). The resultant $\delta^{44/42}$Ca values (+0.90‰, +0.35‰, and -0.50‰, respectively) are in good agreement with previously published values available on GeoRem [22] and their long-term reproducibility (± 2SD) is equal to 0.08‰ (IAPSO, n = 135), 0.10‰ (SRM-915b, n = 15), and 0.05‰ (SRM-1486, n = 172). All analytical results for the $\delta^{44/42}$Ca composition of groundwater samples are provided in the Supplementary Data Tables section. For ease of comparison with other previously published datasets, a conversion calculation to $\delta^{44/40}$Ca is provided in Table 1 of the manuscript. This conversion factor is from Gussone et al., 2016 [29] which assumes a kinetic fractionation coefficient of 2.05, where $\delta^{44/40}$Ca = $\delta^{44/42}$Ca x 2.05.

d. Sr Isotope Analyses

Groundwater Sr isotopic compositions were measured at GEOMAR Helmholtz Center for Ocean Research (Kiel, GER) using a method developed by Krabbenhöft et al. (2009) [30], which involves the chemical separation of every sample twice, once ‘spiked’ using a custom double spike and once ‘unspiked.’ Ion-exchange chromatography was conducted using Eichrom Sr Spec resin in BioRad™ micro bio-spin™ columns, followed by Sr ($\delta^{88/86}$Sr and $^{87}Sr/^{86}Sr$) isotope analysis using a TRITON thermal ionization mass spectrometer (TIMS) (ThermoFisher, Bremen, Germany). The results of the $\delta^{88/86}$Sr measurements are reported in δ-notation relative to the SRM-987 reference standard and calculated as:

$$\delta^{88/86}Sr\ (‰) = [(^{88}Sr/^{86}Sr)_{sample} / (^{88}Sr/^{86}Sr)_{SRM\text{-}987} - 1] \times 1000$$

To correct for session-to-session variations in isotopic ratios, 3 'spiked' and 2 'unspiked' SRM-987 analyses were conducted during each session – alongside every 8 samples. The average measured value of these SRM-987 analyses were compared to their accepted values ($^{88/86}Sr$ = 8.375209, $\delta^{88/86}Sr$ = 0, and $^{87}Sr/^{86}Sr$ = 0.710240) to calculate a correction factor for the session. This correction factor was applied to each sample, resulting in session-corrected values. Reported in Krabbenhoft et al. (2009) [30], the long-term $\delta^{88/86}Sr$ reproducibility of SRM-987 with this method and instrumentation is 0.012 ± 0.044 (2sd). All analytical results for the $\delta^{88/86}Sr$ and $^{87}Sr/^{86}Sr$ composition of groundwater samples are provided in the Supplementary Data Tables section.

e. Ba Isotope Analyses

Groundwater samples were prepared and measured for their Ba isotopic compositions at the NIRVANA Labs at WHOI using the method described by Bates et al. (2017) [31]. Samples were spiked with an appropriate quantity of ^{135}Ba–^{136}Ba double spike so as to achieve a spike- to sample-derived Ba molar ratio of between 1–2. Samples with salinity >5 were co-precipitated with $CaCO_3$ via dropwise addition of Ba-free 1 M Na_2CO_3 solution, dissolved in 6 M HCl and reconstituted in 250 µL of 2 M HCl. Samples with salinity < 5 were evaporated and reconstituted in 250 µL of 2 M HCl. Reconstituted samples were twice passed through columns containing 500 µL of AG 50W-X8 resin; matrix elution used 2 M HCl, while Ba was eluted (and REE retained) with 2 M HNO_3. The resin was discarded after use. Purified samples were reconstituted in 0.5 M HNO_3 to achieve a sample-derived [Ba] of ≈ 20 ng mL^{-1}.

Analyses were conducted at the WHOI Plasma Facility using a ThermoFinnigan Neptune MC-ICP-MS, operated in low resolution mode. Samples were aspirated, desolvated, and introduced into the mass spectrometer using a PFA nebulizer (at a rate of ≈140 µL min^{-1}),

CETAC Aridus II, and as an aerosol in ~1 L Ar min^{-1} containing 3–5 mL min^{-1} admixed N_2, respectively. Samples were analyzed in 30–40 × ≈4.2 s integrations a minimum of two times, up to a maximum of eight. Spiked aliquots of NIST SRM 3104a were measured every fifth analysis; the spike-to-sample ratio of NIST SRM 3104a was adjusted to match bracketing samples and isotopic data reported relative to the nearest four bracketing standards using the δ notation:

$$\delta^{138/134}Ba\ (‰) = [(^{138}Ba/^{134}Ba)_{sample} / (^{138}Ba/^{134}Ba)_{NIST\ SRM\text{-}3104a} - 1].$$

Results for groundwater samples are provided in the section for Supplementary Data Tables. Analytical precision is reported as the greater of either the long-term 2 SD reproducibility (± 0.03 ‰; Horner et al., 2015) or the measured 2 SE obtained from replicate analyses (i.e., where *n* was between 2–8). Accuracy was monitored by processing four aliquots of GEOTRACES SAFe D1 alongside sample unknowns; two aliquots were processed from bottle #591 and two from #596, yielding mean [Ba] and $\delta^{138}Ba$ of 99.0±2.5 nmol kg^{-1} and +0.33±0.03 ‰, and 99.4±2.5 nmol kg^{-1} and +0.33±0.03 ‰, respectively. Results from both bottles are in agreement with previous measurements of SAFe D1 from the NIRVANA Labs (98.7±2.5 nmol kg^{-1} and +0.31±0.03 ‰; Geyman et al., 2019) and elsewhere (99.6 nmol kg^{-1} and +0.27±0.02 ‰; Hsieh & Henderson, 2017).

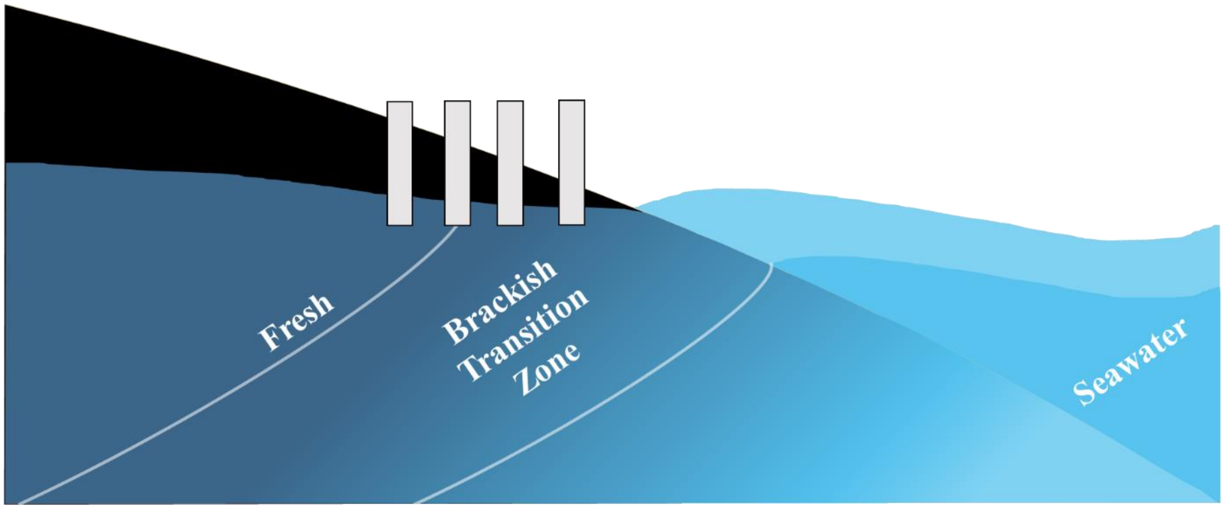

Supplementary Figure 1. Conceptual diagram of the subterranean estuary and sampling scheme, where grey bars represent sampling across a salinity gradient.

Supplementary Table 1. Riverine δ^{138}Ba compilation

River	[Ba] (nM)	δ^{138}Ba (‰)	Discharge (km^3/yr)
Changjiang	432	0.19	688.2
Amazon	144	0.14	5616
Yukon	1467	0.16	339.6
Pearl	209	0.17	336
Sepik	48	0.25	119
Danube	248	0.13	71.6
Lena	90	0.32	525
Colorado	1049	0.3	15.4
Yellow River	1400	0.27	23.2
Rio De Plata	206	0.05	473.2
Discharge weighted average:	246	0.16	-

Supplementary Table 2.	Aquifer type, global riverine, and global groundwater chemical composition		
Aquifer (this study)	Geology	Aquifer Type	Associated Reference
Veraguas Province, Panama	Extrusive volcanics	Extrusive igneous	Beck et al., 2013 [1]
Hawai`i Island, HI, USA	Modern basalt (<1 Ma)	Extrusive igneous	Knee et al., 2010 [2]
Oahu, HI, USA	Quaternary basalt (<2 Ma)	Extrusive igneous	Mayfield, 2013 [3]
Maui, HI, USA	Quaternary basalt (<2 Ma)	Extrusive igneous	Bishop et al., 2015 [4]
Moorea, French Polynesia	1.5 - 2.0 Ma volcanics	Extrusive igneous	Knee et al., 2016 [5]
Flic-en-Flac Lagoon, Mauritius	< 2 Ma basalt	Extrusive igneous	Povinec et al., 2012 [6]
Puerto Morelos, Yucatan, Mexico	Holocene (<12 ka) karstic limestone	Carbonate	Null et al., 2014 [7]
Cape Coral, FL, USA	Pleistocene-Holocene sediments (<2 Ma)	Carbonate	Liu et al., 2014 [8]
Rottnest Island, Australia	Pleistocene-Holocene limestone (<2 Ma)	Carbonate	Bryan et al., 2016 [9]
Venice Lagoon, Venice, Italy	Modern carbonates	Carbonate	Rapaglia et al., 2010 [10]
*Everglades, FL, USA	Modern carbonates with some evaporites	Carbonate	Holmden et al., 2010 [11]
*Carbonate GROUNDWATER average (Florida, Yucatan average)	Modern carbonates with some evaporites	Carbonate	Holmden et al., 2010 [11]
Waquoit Bay, MA, USA	Granitic Glacial Till	Intrusive Igneous	Liu et al., 2017 [8]
Great South Bay, NY, USA	Granitic Glacial Till	Intrusive Igneous	Beck et al., 2007 [12]
Monterey, CA, USA	Mesozoic granites (~180 Ma) and Cenozoic (<66 Ma) sediments	Intrusive Igneous	Lecher et al., 2016 [13]
Kasitsna Bay, AK, USA	Mesozoic metamorphic, volcanic, and igneous intrusive rocks	Intrusive Igneous	Lecher et al., 2015 [14]
Eilat, Israel	Late Precambrian igneous and metamorphics (630 Ma granite, 790 Ma gneiss, 807 Ma Schists)	Intrusive Igneous	This Study
*Bay of Bengal, Bangladesh	Igneous granites and gneisses	Intrusive Igneous	Beck et al., 2013 [1]
*Pamet River, MA, USA	Granitic Glacial Till	Intrusive Igneous	Beck et al., 2013 [1]
*Staten Island, NY, USA	Granitic Glacial Till	Intrusive Igneous	Hogan and Blum, 2003 [15]
Seabright, CA, USA	Quaternary Sediments (<2.5 Ma)	Sedimentary	Lecher et al., 2016 [13]
Angel Island, CA, USA	Altered Graywacke (~100 Ma)	Sedimentary	Null et al., 2012 [16]
St Lucia, South Africa	Mesozoic extrusive volcanics (~180 Ma) and Cretaceous (145-65 Ma) Sandstone	Sedimentary	Moore et al., 2019 [17]
Coffs Harbour, Australia	Lower Permian base rock (slate, schistose sandstone/conglomerate, 250 - 298 Ma) with modern sands	Sedimentary	Tucker et al., 2019 [18]
Patos Lagoon, Brazil	Pleistocene and Holocene (<2 Ma) sandy cap on an alluvial fan on Precambrian shield	Sedimentary	Windom et al., 2003 [19]

*Sampling sites for which only historical data is provided - no new analyses were conducted for this study. Associated reference refers to the study in which the data utilized were published

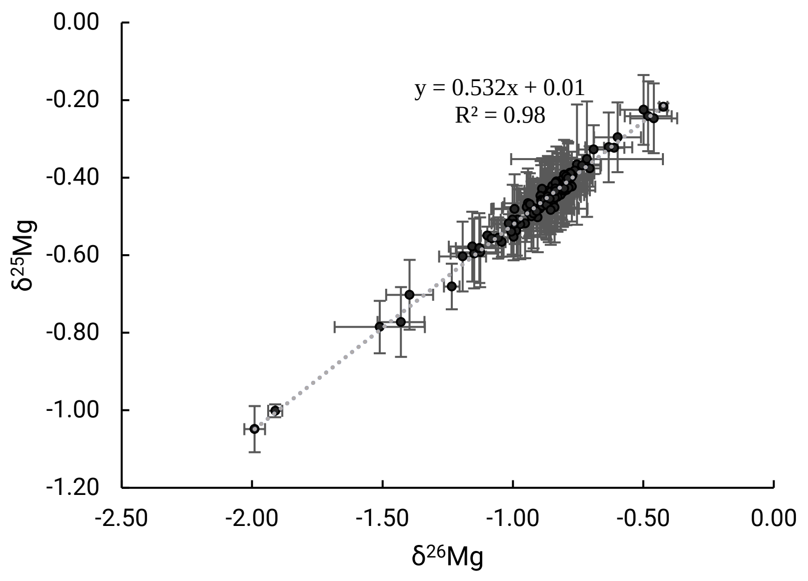

Supplementary Figure 2. Three isotope plot (δ^{26}Mg vs δ^{25}Mg) for all samples measured in this study. Error bars represent 2 standard deviations of the sample average or long-term external precision on the Cambridge-1 standard at Princeton.

Supplementary Data Tables

Supplementary Table 3. | All concentration data collected as a part of this study.

Sample ID	Location	Lithologic 'Aquifer Type'	Salinity	[Li]	[Mg]	[Ca]	[Sr]	[Ba]
				μg L^{-1}	g L-1	g L-1	mg L-1	μg L^{-1}
HBM4	Maui, HI	Extrusive Igneous	33.9	166	1.14	0.39	7.5	43
HBSP3	Maui, HI	Extrusive Igneous	3.5	16	0.07	0.03	0.4	1
Honokowai Well	Maui, HI	Extrusive Igneous	0.0	2	0.02	0.02	0.2	4
HONW	Maui, HI	Extrusive Igneous	0.1	0.31	0.01	0.01	0.1	1
KaBpiez1	Oahu, HI	Extrusive Igneous	24.2	112	0.93	0.31	5.9	10
KaBpiez2	Oahu, HI	Extrusive Igneous	27.5	137	0.94	0.31	5.9	7
KaBpiez3	Oahu, HI	Extrusive Igneous	27.6	141	0.89	0.27	5.5	7
KaBspring1	Oahu, HI	Extrusive Igneous	1.3	4	0.04	0.04	0.4	10
KaBWell	Oahu, HI	Extrusive Igneous	0.0	0.05	0.01	0.02	0.1	2
KONA_104	Kona, HI	Extrusive Igneous	24.8	136	0.95	0.31	5.8	16
KONA_300	Kona, HI	Extrusive Igneous	13.4	60	0.50	0.17	3.1	8

KONA_85	Kona, HI	Extrusive Igneous	24.1	143	0.91	0.28	5.4	12
KONA_92	Kona, HI	Extrusive Igneous	14.0	86	0.58	0.19	3.6	10
KONA_93	Kona, HI	Extrusive Igneous	14.0	73	0.52	0.18	3.3	9
M1	Mauritius	Extrusive Igneous	4.2	18	0.14	0.09	1.2	4
M13	Mauritius	Extrusive Igneous	8.3	46	0.27	0.13	2.0	2
M21	Mauritius	Extrusive Igneous	35.7	180	1.21	0.40	8.2	5
M22 3/24	Mauritius	Extrusive Igneous	34.9	168	1.19	0.41	8.3	6
M22 3/25	Mauritius	Extrusive Igneous	31.9	157	1.09	0.41	7.9	8
M24 3/24	Mauritius	Extrusive Igneous	32.3	152	1.10	0.38	7.3	8
M26 3/24	Mauritius	Extrusive Igneous	32.3	153	1.11	0.37	7.3	6
M4	Mauritius	Extrusive Igneous	36.8	173	1.24	0.43	8.5	8
M5	Mauritius	Extrusive Igneous	36.8	169	1.25	0.43	8.6	8
M6	Mauritius	Extrusive Igneous	5.4	28	0.17	0.10	1.3	5
M9	Mauritius	Extrusive Igneous	36.2	165	1.22	0.42	8.3	6
Th_12	Moorea, French Polynesia	Extrusive Igneous	2.8	43	0.22	0.13	2.0	58
Th_27	Moorea, French Polynesia	Extrusive Igneous	16.6	49	0.40	0.24	6.0	64
Th_42	Moorea, French Polynesia	Extrusive Igneous	15.1		0.00	0.00	0.0	
Th_47	Moorea, French Polynesia	Extrusive Igneous	16.4	46	0.42	0.25	7.3	21
Th_53	Moorea, French Polynesia	Extrusive Igneous	6.0	60	0.22	0.13	2.1	6
Th_60	Moorea, French Polynesia	Extrusive Igneous	14.2		0.55	0.25	5.5	76
Th_62	Moorea, French Polynesia	Extrusive Igneous	21.9		0.81	0.38	8.5	6
LJL227	West Panama	Extrusive Igneous	30.0	118	1.33	0.52	9.5	256
LJL228	West Panama	Extrusive Igneous	0.5	5	0.04	0.02	0.3	15
LJL229	West Panama	Extrusive Igneous	11.5	49	0.30	0.11	2.0	205
LJL230	West Panama	Extrusive Igneous	1.0	5	0.02	0.02	0.1	2
LJL250	West Panama	Extrusive Igneous	5.1	13	0.15	0.20	1.6	48
LJL251	West Panama	Extrusive Igneous	4.2	11	0.12	0.15	1.2	110
V Artisian Well	Venice, Italy	Carbonate	0.7	19	0.04	0.04	0.5	313
VP02	Venice, Italy	Carbonate	12.9	113	0.65	0.33	4.6	43
VP05	Venice, Italy	Carbonate	16.6	104	0.70	0.35	4.8	65
VP07	Venice, Italy	Carbonate	20.4	112	0.80	0.36	5.2	54
VP3	Venice, Italy	Carbonate	14.4	90	0.58	0.29	3.9	51
VP4	Venice, Italy	Carbonate	15.9	84	0.62	0.31	4.2	68
VP-6	Venice, Italy	Carbonate	18.9	111	0.76	0.37	5.3	56
VP8	Venice, Italy	Carbonate	21.3	128	0.93	0.43	6.1	72
Rott1	Rottnest Island, West Australia	Carbonate	24.4	110	0.83	0.34	0.0	62
Rott2	Rottnest Island, West Australia	Carbonate	37.9	184	1.28	0.43	8.4	7
Rott3	Rottnest Island, West Australia	Carbonate	8.0	65	0.25	0.19	0.0	19
Rott4	Rottnest Island, West Australia	Carbonate	0.3	4	0.04	0.05	2.3	2
Rott5	Rottnest Island, West Australia	Carbonate	19.0	74	0.68	0.34	5.0	30
CAL 212	Cape Coral, FL	Carbonate	20.4	21	0.67	1.27	9.3	117
CAL 213	Cape Coral, FL	Carbonate	25.9	36	0.96	0.67	8.9	261
CAL 215	Cape Coral, FL	Carbonate	8.3	49	0.28	0.15	2.2	13

GW 213	Cape Coral, FL	Carbonate	0.4	16	0.03	0.04	2.1	14
FIC_7	Yucatan, Mexico	Carbonate		5	0.32	0.20	4.4	26
FIC_17	Yucatan, Mexico	Carbonate	0.0	2	0.14	0.12	3.2	51
MEX_11	Yucatan, Mexico	Carbonate	39.5	179	1.34	0.46	9.5	19
MEX_63	Yucatan, Mexico	Carbonate	30.6	141	1.04	0.37	7.1	10
MEX_66	Yucatan, Mexico	Carbonate	42.4	181	1.42	0.50	10.2	22
SGD Eilat	Eilat, Israel	Intrusive Igneous	39.7	185	1.33	0.47	9.7	16
MB Academy 1	Monterey, CA	Intrusive Igneous	33.6	160	1.11	0.39	7.8	12
MB Academy 5	Monterey, CA	Intrusive Igneous	27.1	160	1.16	0.41	8.1	18
MB Hopkins 10	Monterey, CA	Intrusive Igneous		304	2.37	0.83	17.7	21
MB Hopkins 11	Monterey, CA	Intrusive Igneous	22.6	161	0.44	0.33	3.9	37
MB Hopkins 15	Monterey, CA	Intrusive Igneous	15.5	134	1.12	0.38	7.5	15
MB Hopkins 16	Monterey, CA	Intrusive Igneous	34.9	152	1.15	0.39	7.9	11
MB Hopkins 18	Monterey, CA	Intrusive Igneous	21.8	159	0.70	0.39	5.7	39
MB Hopkins 19	Monterey, CA	Intrusive Igneous	33.7	149	1.10	0.39	7.7	8
MB Hopkins 39	Monterey, CA	Intrusive Igneous	26.5	150	1.13	0.41	7.8	14
WP 10-5	Southern Brazil	Intrusive Igneous	26.3	206	1.34	0.40	6.4	13
WP 12-5	Southern Brazil	Intrusive Igneous	22.6	155	1.14	0.34	5.6	10
WP 1-5	Southern Brazil	Intrusive Igneous	25.8	166	1.31	0.39	5.9	11
WP 2-5	Southern Brazil	Intrusive Igneous	22.8	156	1.16	0.35	5.7	12
WP 3-S	Southern Brazil	Intrusive Igneous	24.8	172	1.25	0.38	6.0	11
WP 5-5	Southern Brazil	Intrusive Igneous	25.1	171	1.26	0.38	6.1	13
WP 6-5	Southern Brazil	Intrusive Igneous	28.1	201	1.42	0.44	6.8	9
DT1	Waquoit Bay, MA	Intrusive Igneous	0.3	1	0.01	0.01	0.1	1
DT2	Waquoit Bay, MA	Intrusive Igneous	7.6	40	0.27	0.11	1.9	106
DT3	Waquoit Bay, MA	Intrusive Igneous	10.1	40	0.41	0.15	2.7	55
DT4	Waquoit Bay, MA	Intrusive Igneous	17.8	76	0.66	0.22	4.2	31
DT8	Waquoit Bay, MA	Intrusive Igneous	25.6	109	0.95	0.31	6.2	44
DT9	Waquoit Bay, MA	Intrusive Igneous	1.6	8	0.04	0.02	0.3	4
GSB Ba peak	Great South Bay, NY	Intrusive Igneous	30.7	113	1.04	0.35	7.3	173
GSB Fresh	Great South Bay, NY	Intrusive Igneous	0.6	4	0.01	0.02	0.2	25
GSB Mid	Great South Bay, NY	Intrusive Igneous	20.0	102	0.69	0.23	4.4	24
GSB Mid2	Great South Bay, NY	Intrusive Igneous	29.5	140	0.99	0.32	6.5	40
GSB Saline	Great South Bay, NY	Intrusive Igneous	31.7	147	1.08	0.34	7.0	17
AK_102	Kasitsna, AK	Intrusive Igneous	26.2	110	0.92	0.32	6.0	15
AK_115	Kasitsna, AK	Intrusive Igneous	28.9	162	1.04	0.35	6.7	10
AK_131	Kasitsna, AK	Intrusive Igneous	30.2	156	1.06	0.35	7.0	9
AK_15	Kasitsna, AK	Intrusive Igneous	27.9	165	1.07	0.36	7.0	8
AK_38	Kasitsna, AK	Intrusive Igneous	6.3	57	0.17	0.06	1.0	2
AK_48	Kasitsna, AK	Intrusive Igneous	14.5	74	0.47	0.16	2.9	9
AK_49	Kasitsna, AK	Intrusive Igneous	30.4	118	0.85	0.30	5.5	20
AK_75	Kasitsna, AK	Intrusive Igneous	28.7	84	0.39	0.14	2.4	8

Aus 1	East Australia	Sedimentary	2.2	26	0.09	0.16	1.4	55
Aus 10	East Australia	Sedimentary	25.4	122	0.96	0.33	6.1	25
Aus 11	East Australia	Sedimentary	4.4	26	0.20	0.09	1.3	54
Aus 12	East Australia	Sedimentary	4.3	17	0.13	0.08	1.0	23
Aus 13	East Australia	Sedimentary	32.4	181	1.23	0.42	7.9	24
Aus 14	East Australia	Sedimentary	19.7	114	0.77	0.26	4.7	23
Aus 15	East Australia	Sedimentary	11.5	52	0.48	0.18	3.0	30
Aus 16	East Australia	Sedimentary	2.0	12	0.05	0.04	0.4	68
Aus 17	East Australia	Sedimentary	36.3	192	1.28	0.43	8.8	26
Aus 18	East Australia	Sedimentary	35.0	179	1.27	0.42	8.5	47
Aus 2	East Australia	Sedimentary	1.8	25	0.08	0.10	1.1	47
Aus 3	East Australia	Sedimentary	0.4	6	0.01	0.09	0.5	775
Aus 4	East Australia	Sedimentary	0.3	7	0.01	0.07	0.4	32
Aus 5	East Australia	Sedimentary	17.0	74	0.62	0.25	4.1	189
Aus 6	East Australia	Sedimentary	6.0	33	0.21	0.24	2.7	9
Aus 7	East Australia	Sedimentary	0.4	10	0.04	0.06	0.5	8
Aus 8	East Australia	Sedimentary	0.3	3	0.01	0.06	0.3	3
Aus 9	East Australia	Sedimentary	16.5	86	0.66	0.24	4.0	35
MB_31	Santa Cruz, CA	Sedimentary	30.7	178	1.30	0.43	8.7	27
MB_34	Santa Cruz, CA	Sedimentary	9.8	81	0.40	0.14	2.3	14
MB_38	Santa Cruz, CA	Sedimentary	33.6	163	1.25	0.41	8.3	12
MB_56	Santa Cruz, CA	Sedimentary	22.5	129	0.79	0.27	5.0	13
MB_78	Santa Cruz, CA	Sedimentary	10.2	80	0.36	0.12	2.1	8
S.A. ocn	South Africa	Sedimentary	37.0	170	1.24	0.41	8.6	7
SA Fresh	South Africa	Sedimentary	0.2	14	0.03	0.01	0.2	60
SA SL-EM-#3	South Africa	Sedimentary	8.0	57	0.29	0.14	2.1	26
SA SL-EM-#6	South Africa	Sedimentary	7.0	41	0.20	0.08	1.4	7
SFB Al 17 5/10	San Francisco, CA	Sedimentary	17.6	100	0.75	0.23	3.8	29
SFB_2	San Francisco, CA	Sedimentary	25.8	142	1.06	0.36	6.9	30
SFB_22	San Francisco, CA	Sedimentary	22.6	135	0.94	0.33	6.4	22
SFB_27	San Francisco, CA	Sedimentary	25.5	134	0.94	0.33	6.4	36
SFB_39	San Francisco, CA	Sedimentary	24.4	125	0.95	0.33	6.5	37
SFB_72	San Francisco, CA	Sedimentary	26.1	124	0.97	0.34	6.3	39
SFB_85	San Francisco, CA	Sedimentary	9.4	45	0.24	0.12	1.7	21
SFB17 5/10	San Francisco, CA	Sedimentary	17.6	110	0.73	0.25	4.6	30
SFB361009	San Francisco, CA	Sedimentary	17.5	138	0.97	0.35	6.4	31
SFBAI171009	San Francisco, CA	Sedimentary	17.6	132	0.93	0.34	6.2	33

Supplementary Table 4. | All isotope data collected as a part of this study.

Sample ID	δ^7Li	± 2 SD	$\delta^{26/24}$Mg	± 2 SD	$\delta^{44/42}$Ca	± 2 SD	^{87}Sr/^{86}Sr	$\delta^{88/86}$Sr	$\delta^{138/134}$Ba	± 2 SD

	‰	‰	‰	‰	‰	‰		‰	‰	‰
HBM4			-0.75	0.09			0.70931	0.411		
HBSP3	29.7	0.2	-0.99	0.05	0.87	0.07	0.70929	0.421		
Honokowai Well			-1.10	0.03	0.49	0.05	0.70637	0.317		
HONW	26.4	0.1	-0.61	0.04	0.43	0.04	0.70443	0.319		
KaBpiez1	31.7	0.4	-0.88	0.01			0.70928	0.421	0.40	0.04
KaBpiez2					0.87	0.06	0.70929	0.434		
KaBpiez3					0.86	0.06	0.70932	0.432		
KaBspring1			-0.86	0.09	0.55	0.07	0.70727	0.345		
KaBWell	31.4	0.3			0.50	0.03	0.70554	0.390	0.16	0.03
KONA_104			-0.77	0.03	0.99	0.08			0.08	
KONA_300	30.9	0.3	-0.88	0.04	0.92	0.06	0.70922	0.353	0.02	
KONA_85	30.3	0.1	-0.87	0.06	1.01				0.11	
KONA_92			-0.85	0.00	0.88				-0.03	
KONA_93			-0.83	0.01	0.87				0.03	
M1	30.8	0.2	-1.06	0.05	0.62	0.04	0.70901	0.359	-0.18	0.08
M13			-0.89	0.01	0.82	0.09	0.70917	0.368		
M21			-1.08	0.02	0.91	0.05	0.70931	0.398		
M22 3/24			-0.98	0.06	0.89	0.09	0.70931	0.401		
M22 3/25			-0.92	0.08	0.76	0.04	0.70930	0.399		
M24 3/24					0.83	0.10				
M26 3/24			-0.90	0.18	0.84	0.06	0.70931	0.416		
M4					0.87	0.08	0.70933	0.442		
M5			-0.89	0.09	0.84	0.10	0.70931	0.402		
M6					0.74	0.07	0.70904	0.335		
M9	30.0	0.2	-0.94	0.07	0.82	0.09	0.70931	0.397	0.42	0.03
Th_12	29.1	0.2	-1.00	0.04					0.11	
Th_27			-0.83	0.09						
Th_42			-0.72	0.29						
Th_47			-0.83	0.09						
Th_53	28.9	0.1	-0.85	0.09			0.70915	0.385	0.19	
Th_60			-1.02	0.07					0.13	
LJL227			-0.84	0.02	0.78	0.05				
LJL228	27.6	0.1			0.63	0.08	0.70909	0.403	0.36	0.03
LJL229	27.9	0.1	-0.81	0.12	0.83	0.12	0.70908	0.426	0.33	0.03
LJL230			-0.71	0.04						
LJL250			-0.77	0.04			0.70818	0.306		
LJL251			-0.83	0.04			0.70822	0.313		
V Artisian Well	20.7	0.2	-1.43	0.09	0.50	0.01	0.70843	0.267	0.04	0.03
VP02					0.73	0.07	0.70930	0.398		
VP05			-0.79	0.09	0.80	0.10	0.70930	0.405		
VP07			-0.87	0.09	0.78	0.04	0.70930	0.413		

VP3			-0.88	0.09	0.70	0.04	0.70929	0.407		
VP4	27.4	0.2	-0.87	0.09	0.68	0.09	0.70929	0.399	0.10	0.03
VP-6			-0.80	0.09	0.75	0.02	0.70929	0.400		
VP8					0.68	0.08	0.70929	0.399		
Rott1			-0.85	0.10	0.49	0.05	0.70928	0.236		
Rott2					0.91	0.08	0.70933	0.385		
Rott3			-1.51	0.17	0.53	0.08	0.70927	0.215		
Rott4	28.1	0.3	-1.91	0.03	0.53	0.06	0.70926	0.262	0.24	0.06
Rott5	30.3	0.1	-0.85	0.02	0.70	0.05	0.70932	0.313	0.09	0.04
CAL 212	18.2		-0.97	0.09	0.37	0.03	0.70917	0.318	-0.13	0.03
CAL 213			-0.92	0.12	0.52	0.06	0.70915	0.334	0.09	0.03
CAL 215	34.1		-0.86	0.09	0.75	0.05	0.70914	0.352		
GW 213	23.1	0.0	-1.99	0.04	0.43	0.06	0.70869	0.236	0.46	0.03
FIC_17									0.24	0.04
MEX_11									0.43	
MEX_63	30.1	0.1	-0.78	0.05	0.93	0.11			0.30	
MEX_66					0.93	0.07			0.30	
SGD Eilat	28.5	0.2			0.94	0.07			0.49	0.03
MB Academy 1					0.93	0.08				
MB Academy 5			-0.79	0.09	0.95	0.09				
MB Hopkins 10			-0.86	0.09	0.88	0.05	0.70934	0.462		
MB Hopkins 11	19.9	0.3	-0.60	0.09	0.71	0.16	0.70932	0.395	0.23	0.03
MB Hopkins 15	17.1	0.1	-0.91		0.69	0.08	0.70935	0.447		
MB Hopkins 16	31.5	0.1	-0.89	0.09						
MB Hopkins 18			-0.83	0.09					0.39	0.03
MB Hopkins 19			-0.77	0.09	0.92	0.04				
MB Hopkins 39					0.82	0.09				
WP 10-5	29.5	0.1			0.93	0.06				
WP 12-5	30.3	0.3			0.91	0.04				
WP 1-5	30.7	0.3			0.88	0.06	0.70936	0.440		
WP 2-5	31.1	0.2			0.97	0.09	0.70936	0.477		
WP 3-S	30.1	0.2			0.86	0.09			0.25	0.05
WP 5-5	30.6	0.4	-1.01	0.03	0.78	0.05			0.04	0.05
WP 6-5	30.2	0.2	-0.86	0.09	0.87	0.11			0.07	0.06
DT1	23.6	0.1	-0.95	0.09	0.57	0.08			0.04	0.04
DT2	49.4	0.3	-0.90	0.09	0.82	0.10	0.70934	0.411	-0.28	0.03
DT3	43.2	0.2	-0.87	0.05	0.83	0.03	0.70935	0.430		
DT4	39.7	0.2	-1.13	0.09	0.90	0.01				
DT8	32.5	0.4	-0.80	0.09	0.87	0.09				
DT9	28.2	0.1	-0.79	0.02	0.88	0.18				
GSB Ba peak	34.4	0.1	-0.85	0.09	0.93	0.04	0.70931	0.376	0.07	0.03
GSB Fresh	33.3	0.0	-1.23	0.03	0.49	0.04			-0.01	0.07
GSB Mid	30.4	0.4	-0.83	0.09	0.85	0.10	0.70933	0.413		

GSB Mid2	30.3	0.0	-0.83	0.07	0.87	0.08	0.70933	0.415		
GSB Saline	29.8	0.3	-0.78	0.06	0.82	0.06	0.70930	0.387		
AK_102	29.3	0.4	-0.82	0.09					0.28	
AK_115			-0.82	0.09	1.05				0.39	
AK_131			-0.82	0.09	0.99				0.40	
AK_15			-0.81	0.09	1.01				0.37	
AK_38	30.5	0.1	-0.85	0.09						
AK_48	29.7	0.1	-0.91	0.16	0.95				0.30	
AK_49									0.22	
AK_75			-0.86	0.09	0.94		0.70923	0.358	0.25	
Aus 1	16.9		-0.89	0.09	0.52	0.02	0.70927	0.270		
Aus 10	30.9	0.2	-0.87	0.09	0.80	0.08				
Aus 11	16.6				0.67	0.06	0.70933	0.367		
Aus 12	16.6		-0.88	0.09	0.39	0.10	0.70929	0.288		
Aus 13			-0.95	0.09	0.86	0.04	0.70932	0.388		
Aus 14					0.85	0.07				
Aus 15	30.8	0.3	-1.15	0.09	0.76	0.10	0.70932	0.390		
Aus 16			-0.94	0.09			0.70929	0.298		
Aus 17			-0.98	0.09	0.98	0.00				
Aus 18			-0.99	0.09	0.89	0.01				
Aus 2			-0.98	0.09	0.52	0.06	0.70929	0.340		
Aus 3	18.4	0.1	-1.40	0.09	0.46	0.09	0.70931	0.343	-0.01	0.04
Aus 4			-1.19	0.09	0.41	0.01	0.70929	0.293		
Aus 5	30.1	0.2	-0.48	0.09	0.79	0.11	0.70931	0.391		
Aus 6			-1.16	0.09	0.49	0.06	0.70927	0.275		
Aus 7	21.2	0.2	-0.87	0.09	0.53	0.02	0.70930	0.310		
Aus 8	19.3	0.2	-1.13	0.09	0.75	0.05				
Aus 9			-0.83	0.09			0.70931	0.369		
MB_31	30.7	0.3			0.93	0.03			0.26	
MB_34			-0.81	0.09	0.87	0.06			0.28	
MB_38	31.4	0.2			0.85	0.09	0.70932	0.388	0.36	
MB_56					0.94	0.08			0.25	
MB_78			-0.93	0.09	0.89	0.09	0.70938	0.423	0.29	
S.A. ocn	30.6	0.2			0.93	0.07	0.70932	0.423		
SA Fresh	17.5	0.0	-0.81	0.09	0.95	0.08			0.25	0.03
SA SL-EM-#3	35.5	0.2	-0.79	0.09	0.75	0.19	0.70936	0.461	0.15	0.03
SA SL-EM-#6	30.5	0.2	-0.86	0.06	0.85	0.06	0.70932	0.398		
SFB AI 17 5/10	30.3	0.4	-0.84	0.09	0.85	0.07				
SFB_2	30.7	0.6	-0.92	0.09	0.72		0.70929	0.345	0.09	
SFB_22			-0.81	0.09	0.85	0.10	0.70928	0.365	0.25	
SFB_27			-0.83	0.09	0.95				0.14	
SFB_39			-1.00	0.09	0.88	0.07	0.70914		0.18	
SFB_72	31.0	0.4			0.83		0.70914	0.391	0.08	

SFB_85	30.0	0.2	-1.00	0.02	0.84	0.11	0.70921	0.348
SFB17 5/10			-0.50	0.09			0.70922	0.323
SFB361009			-0.82	0.02	0.91	0.05		
SFBAI171009					0.86	0.05		

Supplementary References

1. Beck, A., Charette, M., Cochran, J. K., Gonneea, M. & Peucker-Ehrenbrink, B. Dissolved strontium behavior in the subterranean estuary and implications for the Sr mass balance and isotope budget of the global ocean. *Geochim. Cosmochim. Acta* **117**, 33–52 (2013).
2. Knee, K., Street, J. H., Grossman, E. G. & Paytan, A. Nutrient inputs to the coastal ocean from submarine groundwater discharge in a groundwater-dominated system: Relation to land use (Kona coast, Hawaii, U.S.A.). *Limnol. Oceanogr.* **55**, 1105–1122 (2010).
3. Mayfield, K. K. A summary of the submarine groundwater discharge (SGD) in Kahana Bay: spatial and intra-daily variability. (University of Hawaii at Manoa, 2013).
4. Bishop, J. M., Glenn, C. R., Amato, D. W. & Dulai, H. Effect of land use and groundwater flow path on submarine groundwater discharge nutrient flux. *J. Hydrol. Reg. Stud.* **11**, 194–218 (2017).
5. Knee, K. L., Crook, E. D., Hench, J. L., Leichter, J. J. & Paytan, A. Assessment of Submarine Groundwater Discharge (SGD) as a Source of Dissolved Radium and Nutrients to Moorea (French Polynesia) Coastal Waters. *Estuaries and Coasts* **39**, 1651–1668 (2016).
6. Povinec, P. P. *et al.* Isotopic, geophysical and biogeochemical investigation of submarine groundwater discharge: IAEA-UNESCO intercomparison exercise at Mauritius Island. *J. Environ. Radioact.* **104**, 24–45 (2012).
7. Null, K. A., Knee, K. L., Crook, E. D. & Sieyes, N. R. De. Composition and fluxes of submarine groundwater along the Caribbean coast of the Yucatan Peninsula. *Coast. Shelf Res.* **77**, 38–50 (2014).
8. Liu, Q. *et al.* Carbonate system biogeochemistry in a subterranean estuary: Waquoit Bay, USA. *Geochim. Cosmochim. Acta* **203**, 422–439 (2017).
9. Bryan, E., Meredith, K. T., Baker, A., Post, V. E. A. & Andersen, M. S. Island groundwater resources, impacts of abstraction and a drying climate: Rottnest Island, Western Australia. *J. Hydrol.* **542**, 704–718

(2016).

10. Rapaglia, J. *et al.* Investigation of residence time and groundwater flux in Venice Lagoon: Comparing radium isotope and hydrodynamical models. *J. Environ. Radioact.* **101**, 571–581 (2010).

11. Holmden, C., Papanastassiou, D. A., Blanchon, P. & Evans, S. $\delta^{44/40}$Ca variability in shallow water carbonates and the impact of submarine groundwater discharge on Ca-cycling in marine environments. *Geochim. Cosmochim. Acta* **83**, 179–194 (2012).

12. Beck, A. J., Rapaglia, J. P., Cochran, J. K., Bokuniewicz, H. J. & Yang, S. Submarine groundwater discharge to Great South Bay, NY, estimated using Ra isotopes. *Mar. Chem.* **109**, 279–291 (2008).

13. Lecher, A. L., Fisher, A. T. & Paytan, A. Submarine groundwater discharge in Northern Monterey Bay, California: Evaluation by mixing and mass balance models. *Mar. Chem.* **179**, 44–55 (2016).

14. Lecher, A. L. *et al.* Methane transport through submarine groundwater discharge to the North Pacific and Arctic Ocean at two Alaskan sites. *Limnol. Oceanogr.* **61**, S344–S355 (2016).

15. Hogan, J. F. & Blum, J. D. Boron and lithium isotopes as groundwater tracers: A study at the Fresh Kills landfill, Staten Island, New York, USA. *Appl. Geochemistry* **18**, 615–627 (2003).

16. Null, K. A. *et al.* Submarine Groundwater Discharge-Derived Nutrient Loads to San Francisco Bay: Implications to Future Ecosystem Changes. *Estuaries and Coasts* **35**, 1299–1315 (2012).

17. Moore, W. S., Humphries, M. S., Benitez-Nelson, C. R., Pillay, L. & Higgs, C. Transport of Radium and Nutrients Through Eastern South African Beaches. *J. Geophys. Res. Ocean.* **124**, 2010–2027 (2019).

18. Tucker, J. P., Santos, I. R., Davis, K. L. & Butcher, P. A. Whale carcass leachate plumes in beach groundwater: A potential shark attractant to the surf? *Mar. Pollut. Bull.* **140**, 219–226 (2019).

19. Windom, H. & Niencheski, F. Biogeochemical processes in a freshwater-seawater mixing zone in permeable sediments along the coast of Southern Brazil. *Mar. Chem.* **83**, 121–130 (2003).

20. Magna, T., Wiechert, H. & Halliday, A. Low-blank isotope ratio measurement of small samples of lithium using multiple-collector ICPMS. *Int. J. Mass Spectrom.* **239**, 67–76 (2004).

21. Flesch, G. D., Anderson, A. R. & Svec, H. J. A secondary isotopic standard for ^{6}Li/^{7}Li determinations. *Int. J. Mass Spectrom. Ion Phys.* **12**, 265–272 (1973).

22. Jochum, K. P. *et al.* GeoReM: A New Geochemical Database for Reference Materials and Isotopic Standards. *Geostand. Geoanalytical Res.* **29**, 333–338 (2005).

23. Blättler, C. L., Miller, N. R. & Higgins, J. A. Mg and Ca isotope signatures of authigenic dolomite in siliceous deep-sea sediments. *Earth Planet. Sci. Lett.* **419**, 32–42 (2015).

24. Galy, A. *et al.* Magnesium isotope heterogeneity of the isotopic standard SRM980 and new reference materials for magnesium-isotope-ratio measurements. *J. Anal. At. Spectrom.* **18**, 1352–1356 (2003).

25. Higgins, J. A. & Schrag, D. P. Constraining magnesium cycling in marine sediments using magnesium isotopes. *Geochim. Cosmochim. Acta* **74**, 5039–5053 (2010).

26. Romaniello, S. J. *et al.* Fully automated chromatographic purification of Sr and Ca for isotopic analysis. *J. Anal. At. Spectrom.* **30**, 1906 (2015)

27. Eisenhauer, A. *et al.* Calcium isotope ratios in blood and urine: A new biomarker for the diagnosis of osteoporosis. *Bone Reports* **10**, 100200 (2019).

28. Coplen, T. B. *et al.* Compilation of minimum and maximum isotope ratios of selected elements in naturally occurring terrestrial materials and reagents. *U.S. Dep. Inter. U.S. Geol. Surv. Water-Resources Investig. Rep.* **01**–**4222**, 1–76 (2002).

29. Gussone, N. *et al. Calcium Stable Isotope Geochemistry*. *Advances in Isotope Geochemistry* (Springer, 2016).

30. Krabbenhöft, A. *et al.* Determination of radiogenic and stable strontium isotope ratios ($^{87}Sr/^{86}Sr$; $\delta^{88/86}Sr$) by thermal ionization mass spectrometry applying an $^{87}Sr/^{84}Sr$ double spike. (2009).

31. Bates, S. L. *et al.* Barium isotopes reveal role of ocean circulation on barium cycling in the Atlantic. *Geochim. Cosmochim. Acta* **204**, 286–299 (2017).

32. Luijendijk, E., Gleeson, T. & Moosdorf, N. Fresh groundwater discahrge insignificant for the world's oceans but important for coastal ecosystems. *Nature Communications* 11 (1), 1–12 (2020).

33. Zhou, Y., Sawyer, A. H., David, C. H. & Famiglietti, J. S. Fresh submarine groundwater discharge to the near-global coast. *Geophys. Res. Lett.* **46**, 1–9 (2019).

34. Zektser, I. S. *Groundwater as a component of the environment*. (Springer, 2006).

35. Gibbs, M. T. & Kump, L. R. Global chemical erosion during the Last Glacial Maximum and the present: Sensitivity to changes in lithology and hydrology. *Paleoceanography* **9**, 529–543 (1994).

36. Hartmann, J. & Moosdorf, N. The new global lithological map database GLiM: A representation of rock properties at the Earth surface. *Geochemistry, Geophys. Geosystems* **13**, (2012).
